# Short-Term Observation of Ultrasonic Cyclocoagulation in Chinese Patients with End-Stage Refractory Glaucoma: A Retrospective Study

**DOI:** 10.1155/2018/4950318

**Published:** 2018-09-06

**Authors:** Dongpeng Hu, Shu Tu, Chengguo Zuo, Jian Ge

**Affiliations:** State Key Laboratory of Ophthalmology, Zhongshan Ophthalmic Center, Sun Yat-sen University, 54 Xianlie Road, Guangzhou 510060, China

## Abstract

**Purpose:**

To assess the efficacy and safety of HIFU-based ultrasonic cyclocoagulation in Chinese patients with end-stage refractory glaucoma.

**Method:**

Patients were recruited consecutively from May 2016 to May 2017 in the Zhongshan Ophthalmic Center. Ultrasonic cyclocoagulation was performed on every patient, using the EyeOP1 ultrasound emitting device. Return visits were set at 1 day, 7 days, 1 month, and 3 months after the treatment. An intraocular pressure (IOP) reduction of ≥20% while IOP ≥ 5 mmHg was deemed as success. Mean IOP change was assessed. Efficacy of two modes (6 sectors and 8 sectors) was also compared. Complications were recorded for safety evaluation.

**Results:**

61 eyes were treated in this study. The baseline IOP (mean ± SD) was 41.11 ± 10.65 mmHg. The percentage of IOP reduction after treatment was 29.2%, 43.2%, 34.8%, and 23.1% at 1 day, 7 days, 1 month, and 3 months, respectively. Overall success rate at 3 months was 50.0% (26/52). No significant difference was found between the 6 sectors group and the 8 sectors group in terms of the success rate (48.6% vs. 52.9%, *p*=0.768) as well as IOP reduction (*p*=0.417) at 3 months. Primary angle-closure glaucoma (PACG) had the highest success rate (80.0%, 12/15). Scleral thinning existed in 12 eyes, among which 2 developed hypotony (2 mmHg and 3 mmHg). Average pain score decreased massively compared with baseline data.

**Conclusion:**

With high percentage of IOP reduction and a good safety profile observed in our study, HIFU-based ultrasonic cyclocoagulation might become a promising alternative to cyclodestructive methods. Long-term efficacy and safety need further assessment. The study was registered with Chinese Clinical Trial Registry (http://www.chictr.org.cn; Registration number: ChiCTR-OOC-17014028).

## 1. Introduction

High-intensity-focused ultrasound (HIFU) technology is being used to destroy target tissue with HIFU focused onto a small point, where the temperature will go up critically due to thermal effect of ultrasound [1]. With great accuracy, it can effectively avoid damage to the surrounding tissue. HIFU has been widely adopted in the treatment of prostate cancer [2], breast cancer [3], and liver cancer [4]. The application of this technology in glaucoma can be traced back to the 1980s. By coagulating part of the ciliary body, whose epithelium can secrete aqueous humor, HIFU-based ultrasonic cyclocoagulation is regarded as a potential way to reduce intraocular pressure (IOP). The 1-year success rate of ultrasonic cyclocoagulation reported in some literature was about 50% in 1980s [5]. However, due to technical limitations at that time, the ultrasound emitting device was somewhat bulky and complicated to operate. IOP spikes (or major IOP increases) and scleral thinning often happened after the procedure. Occurrence of other severe complications such as scleral perforation, severe hypotony, or phthisis after the treatment was also hard to avoid. Thus, the treatment was gradually abandoned two decades ago [6].

Thanks to advances in technology, ultrasonic cyclocoagulation has made a comeback in recent years. With a miniaturized probe and intelligent operating system, ultrasonic cyclocoagulation can be performed much easily and safely on the eye [7]. Florent Aptel was the first to use miniaturized HIFU device for the treatment of advanced refractory glaucoma, reporting a 1-year success rate of 10/12, and a mean IOP drop of 33.9% [8]. A multicenter clinical study published in 2016 showed that 30 open-angle glaucoma patients without filtering surgery history had a 1-year success rate of 63% (30% mean IOP reduction) after ultrasonic cyclocoagulation and did not present serious complications, suggesting that it is a safe and effective method to reduce IOP [9].

However, because there is a clear distinction between China and European countries in terms of glaucoma types, for example, PACG accounts for about 50% of glaucoma types in China [10] while only 9% [11] in Europe, it cannot be extrapolated that the therapeutic effect of ultrasonic cyclocoagulation still remain the same for Chinese. Although this treatment has been officially approved by China Food and Drug Administration (CFDA) for glaucoma treatment last year, there is no publication regarding the effect of ultrasonic cyclocoagulation in Chinese patients until now. Therefore, in this study, we reviewed records of patients with end-stage glaucoma who underwent ultrasonic cyclocoagulation in the Zhongshan Ophthalmic Center from May 2017 to September 2017, assessing the clinical outcomes of this treatment.

## 2. Patients and Methods

### 2.1. Patients

Glaucoma patients who received ultrasonic cyclocoagulation in the Zhongshan Ophthalmic Center were consecutively recruited from May 2017 to September 2017. The specific inclusion criteria were as follows: (1) end-stage glaucoma according to the Glaucoma Severity Staging (GSS) system [12], (2) IOP ≥ 21 mmHg despite the use of glaucoma medications, and (3) age >18 years and <90 years. Patients were excluded if they met the following criteria: (1) previous cyclophotocoagulation or other cyclodestructive surgery, (2) the presence of glaucoma drainage device, (3) ocular infection, (4) other diseases that can affect intraocular pressure (such as choroidal detachment and subluxation of the lens), or (5) pregnancy or serious systemic disease.

### 2.2. HIFU Device

EyeOP1 device developed by Eye Tech Care company was used in the study. The device consists of three major parts, namely, the control module, coupling cone, and ultrasound emitting probe. Before the treatment, some parameters should be set in the control module. After routine surgical disinfection and topical anesthesia, the coupling cone is firmly attached to the eye via vacuum aspiration, making sure that the position of the pupil is right at the center. Later the ultrasound-emitting probe is placed in the coupling cone, using sterile saline as a coupling agent and cooling agent. After all these steps, treatment procedure can be started. In this study, 8 seconds (2 W) of ultrasound exposure time per sector (6 sectors in total on the probe) and a 20-second interval (sequential sector activation during treatment) were applied. The entire procedure lasts 2 minutes and 28 seconds. In this article, there were two modes (6 sectors and 8 sectors activated) being used for the treatment. 43 patients received 6-sectors activated modality in the early recruitment, while the following patients (18) were treated with 8-sectors, activated, ultrasonic cyclocoagulation. The 8 sectors mode can be achieved easily by rotating the probe to another position. Detailed structure and operation instructions were mentioned in some relevant literature [7, 9].

### 2.3. Methods

This study was approved by the independent Institute Research Ethics Committee at Zhongshan Ophthalmic Center (ZOC, Guangzhou, P. R. China), and written consents were obtained from all participants. Before the treatment, the following baseline information was collected: best-corrected visual acuity, automatic optometry, slit-lamp biomicroscopy with gonioscopy, Goldmann applanation tonometry with three measurements (obtained at 8 am to 9 am), ultrasound pachymetry, and ultrasound biomicroscopy (UBM). Here, UBM is aimed at measuring the diameter of the circle where the ciliary processes are placed, determining the optimal size of the ultrasound emitting probe (10 mm, 11 mm, 12 mm, and 13 mm) for individuals. To be specific, four cross-sections of UBM scan (0°–180°, 45°–225°, 90°–270°, and 135°–315°) were taken into account to calculate the average diameter. All procedures were performed by a senior doctor (Jian Ge) with the assistance of a resident (Shu Tu). Posttreatment follow-up contain corresponding examination data, which were recorded at 1 day, 7 days, 1 month, and 3 months after the treatment. At each visit, photographs of the anterior segment were taken for comparison. One ophthalmologist (Chengguo Zuo) from glaucoma department was in charge of the eye examination and data recording. The number of ocular hypotensive agents was included for analysis. In addition, we used the 0–10 numeric rating scale (NRS) to record the severity of local pain at each follow-up, strictly following the instructions of NRS practice [13].

### 2.4. Outcome Evaluation

In terms of efficacy, the success rate and mean IOP reduction after the treatment were calculated. Success was defined as IOP reduced by ≥20% but still ≥5 mmHg (despite the presence of ocular hypotensive agents), consistent with some related literature [8, 9, 14–20].

Safety assessment depended on the occurrence of posttreatment complications, including pain, bleeding, vision acuity decrease, scleral thinning, hypotony, choroidal detachment, retinal detachment, and phthisis.

### 2.5. Statistical Analysis

Line chart and scatter plot were adopted to present mean IOP trend and individual IOP change, respectively. *W* test was used to analyze the normality of data. Student's *t*-test and analysis of variance were used to compare means, and chi-squared tests or Fisher's exact tests were used for the analysis of dichotomous variables like success rate. Statistical significance was set at *p* < 0.05. Statistical software (SPSS version 17.0; SPSS Inc., Chicago, IL, USA) was used for data analysis.

## 3. Results

### 3.1. Patient Characteristics

The characteristics of 61 glaucoma patients (43 in 6 sectors group/18 in 8 sectors group) were listed in Table 1. Nine of them (9/61, 14.8%) were lost to follow-up at 3 months. Only one eye underwent ultrasonic cyclocoagulation for each patient. One subject received cyclophotocoagulation during follow-up and was seen as failure at 3 months (9 mmHg). Patients could be divided into four groups in our study: primary open-angle glaucoma (POAG) (10/61; 16.4%), primary angle closure glaucoma (PACG) (18/61; 29.5%), neovascular glaucoma (NVG) (29/61; 47.5%), and traumatic glaucoma (4/61; 6.6%). 55 patients presented no light perception visual acuity, while the rest (6 patients) ranged from finger count to light perception.

### 3.2. IOP Trend for all Patients

**Figure 1:**
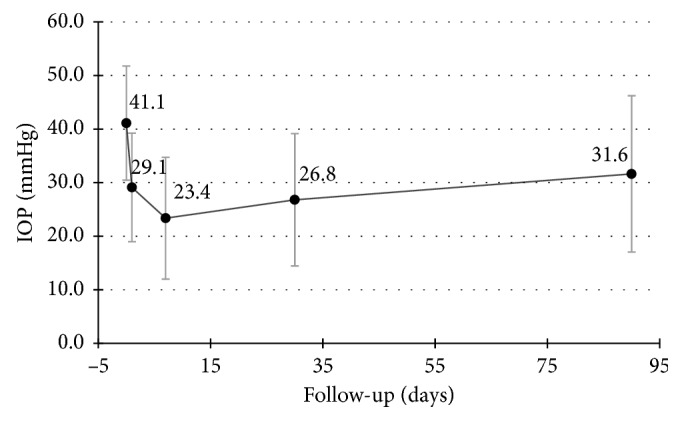
Mean IOP levels at 1 day, 7 days, 1 month, and 3 months after the treatment. Error bars represent standard deviation.

**Figure 2:**
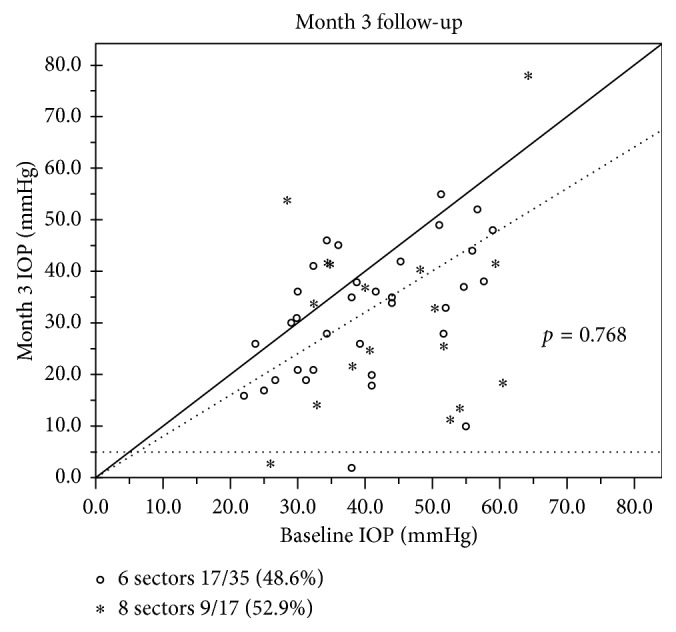
Scatter diagram of IOP changes at 3 months. 50% of cases located in the triangular area (below the oblique dash line *y* = 0.8*x* and above the horizontal dash line *y* = 5) were seen as success cases after the treatment because the IOP reduction was more than 20% but no less than 5 mmHg.

The baseline mean IOP ± SD (standard deviation) was 41.11 ± 10.65 mmHg (Figure 1). The percentage of IOP reduction after the treatment was 29.2% (29.11 ± 10.13 mmHg), 43.2% (23.37 ± 11.37 mmHg), 34.8% (26.79 ± 12.35 mmHg), and 23.1% (31.63 ± 14.59 mmHg) at 1 day, 7 days, 1 month, and 3 months, respectively. Success rate at 3 months was 50.0% (26/52) (Figure 2). The greatest IOP reduction was noted at 7 days. Thereafter, IOP increased slowly while no plateau appeared during follow-up.

### 3.3. IOP Comparison between 6 Sectors and 8 Sectors Groups

**Figure 3:**
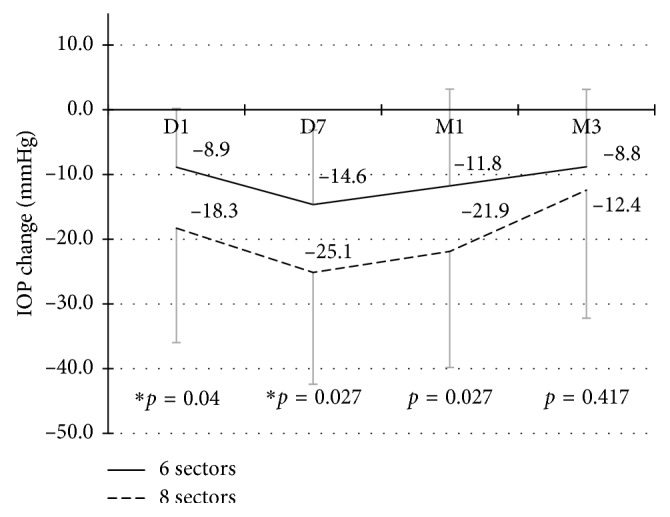
IOP reduction comparison between 6 sectors group and 8 sectors group.

There was no significant difference between the 6 sectors and 8 sectors groups in terms of age (*p*=0.130), diagnosis (*p*=0.867), and surgery history (Table 1). No significant difference (*p*=0.241) of baseline IOP levels was revealed between the two groups (40.07 ± 10.18 mmHg vs. 43.59 ± 11.62 mmHg) (Table 2). However, when it came to mean IOP reduction change (Figure 3), we found that the extent of IOP reduction was much more dramatic in the 8 sectors group at the early stage but not at 3 months (8.81 ± 11.96 mmHg vs. 12.42 ± 19.79, *p*=0.417). In addition, no significant difference of the success rate (48.6% vs. 52.9%, *p*=0.768) was found between the two groups at 3 months (Figure 2).

### 3.4. Different Outcomes among Glaucoma Types

We then conducted subgroup analyses and found out that there was difference in success rate among glaucoma types (*p*=0.013). Interestingly, PACG had the greatest percentage of IOP reduction (36.1%) and the highest success rate (12/15; 80.0%), while NVG seemed to be less responsive to treatment (18.6% of IOP reduction) with the success rate of only 29.2% at 3 months. The success rate for POAG and traumatic glaucoma was 55.6% (5/9) and 50.0% (2/4), respectively. Those who had previous glaucoma surgery (12/52; 11 had a history of trabeculectomy, and 1 had anterior chamber paracentesis) presented a success rates of 75.0% (9/12). For detailed figures across two modalities (6 sectors and 8 sectors), please refer to Table S1. There was no significant difference between patients with and without glaucoma surgery before (17/40; 42.5%) (*p*=0.100; *χ* ^2^ continuity correction) (Table 3).

### 3.5. Safety

The intraoperative and postoperative complications are shown in Table 4. The minor complications were pain, conjunctival hyperemia, anterior chamber reaction, keratic precipitates, bulbar conjunctival edema, and subconjunctival hemorrhage. Most of the signs disappeared within 1 month. Twelve subjects had scleral thinning during follow-up. Typical photographs of anterior segment at each visit and scleral thinning are shown in Supplementary Figure S1. One case showed astigmatism increased from −0.5 diopters to −4.5 diopters. Two patients developed hypotony (2 mmHg and 3 mmHg) (Figure 2), and one of them even presented retinal detachment at last follow-up (Figure S1). However, without b-ultrasound examination of the fundus before the treatment, we had not been able to rule out a possibly preexistent retinal detachment because of the serious cataract. When it came to visual acuity, we found no obvious deterioration due to treatment among six patients with CF-LP VA (Table S2).

### 3.6. Pain Score Decreased Massively

**Figure 4:**
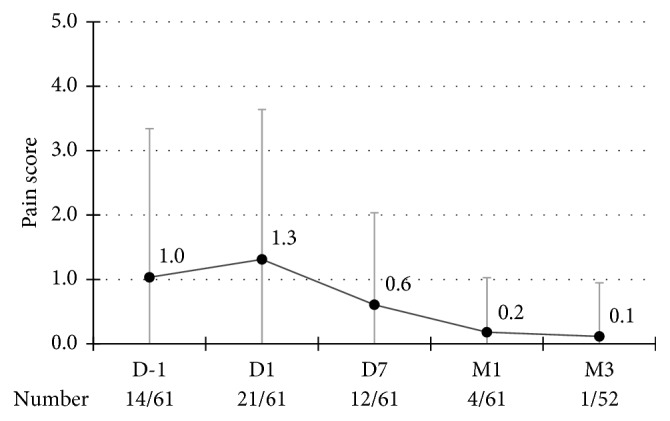
Average pain score and the number of patients suffering local pain at return visits.

All patients involved in our study were end-stage glaucoma patients with severely damaged visual function. A remarkable feature of these patients was the complaint of local pain due to uncontrollable high IOP, in spite of maximal hypotensive agents being administered. In this study, the baseline pain score on average was 1.0, with 14 patients presenting local pain. Although on the day after the treatment, both numbers rose slightly (1.3 points; 21), downward trend was conspicuous afterward. At 3 months, the average pain score was only 0.1 points, and the number of patients with local pain fell to 1 (Figure 4). Statistical significance was detected between baseline and 3 months' average pain score (*p*=0.002). Corneal edema existed in about half of patients complaining of local pain (Table S3).

## 4. Discussion

In this study, the efficacy and safety of HIFU-based ultrasonic cyclocoagulation was observed among end-stage glaucoma patients in China within a 3-month follow-up. The results showed that the mean IOP was decreased by 24.3%, and the success rate was 50.0% at 3 months after the treatment. There was no difference between the 6 sectors and 8 sectors groups with respect to the extent of IOP reduction at last visit. Subgroup analysis indicated that PACG had the highest success rate (80.0%) and largest percentage of IOP reduction (36.1%). However, NVG appeared to be the type that had the lowest response rate, with a success rate of 29.2% (7/24) and only 18.6% of IOP reduction at 3 months. Complications, such as scleral thinning and hypotony, were noticed in our study. The number of patients suffering from local pain and average pain score were reduced significantly at last follow-up.

At present, it is believed that the mechanism of reducing IOP by ultrasonic cyclocoagulation is mainly depending on the destruction of the nonpigmented epithelium of the ciliary body, directly influencing the production of aqueous humor. Besides, due to the shrinkage of the ciliary body after sonification, the potential space between the ciliary body and the sclera is enlarged, leading to increased outflow of aqueous humor through supraciliary and suprachoroidal space [16, 21]. In vivo study also revealed an increase of intrascleral hyporeflective spaces and conjunctival microcysts 1 month after the treatment, indicating an enhanced transscleral AH outflow [22].

The percentage of IOP reduction (24.3%) of our study remained close to the corresponding data (about 25% at 3 months) in some foreign studies [8, 9, 18, 23], and the success rate (50.0%) was only slightly lower. For failed cases with IOP reduction <20%, it is universally believed that the amount of ciliary body coagulated is insufficient, and the remaining normal epithelium will have a compensatory secretion of aqueous humor. Also, it should be taken into account that the increased transscleral outflow after insonification may decrease to some extent over time [22]. For patients with no obvious IOP reduction after treatment, some found that a secondary ultrasonic cyclocoagulation might achieve satisfactory results [23], suggesting that even if no response at the first time, repeated treatment may still be effective.

It was assumed that higher IOP reduction would be seen in the 8 sectors group, for more ciliary processes were considered to be ablated than that in the 6 sectors group. The results turned out to be a little confusing that no statistical differences in IOP reduction and success rate were present between the two groups at 3 months. Only a higher response rate of the 8 sectors group was revealed in the early follow-up. Perhaps, it is because the sample size was not big enough to demonstrate the difference.

Interestingly, we found that PACG did much better than other types of glaucoma, showing a success rate of 80% and mean IOP reduction of 36.1%. Although the mechanism is unknown, it is undoubtedly an encouraging news for Chinese glaucoma patients with a high proportion of PACG.

According to the results of multicenter clinical studies in foreign countries [9, 17, 18], minor complications were completely resolved within 1 month, and no complications such as secondary cataract, subluxation of the lens, and phthisis were observed. Denis et al. [18] (52 patients) reported one case of hypotony with choroidal detachment, one of macular edema, and six of visual acuity reduced >2 lines. Aptel et al. [9] recruited 30 patients and found that two patients developed IOP spikes (increased more than 10 mmHg within 7 days), one case had macular edema, one scleral thinning, and one astigmatism increased more than 1 diopter. In this study, minor complications basically disappeared within 1 month. 12 cases showed scleral thinning, and 2 developed hypotony (2 mmHg and 3 mmHg).

For end-stage glaucoma patients, local pain has a serious impact on the quality of life. It could be caused by uncontrolled IOP as well as corneal edema due to decompensation of corneal endothelium as a result of long-term high IOP. Although the overall success rate was only 50%, it was rather surprising that the pain score reduced massively, which, to some extent, was in line with the goal of treatment for patients with advanced glaucoma.

Compared with other cyclodestructive surgery, ultrasonic cyclocoagulation, as a relatively new method, showed no obvious advantage regarding efficacy for the moment. Taking IOP ≤ 21 mmHg as the success standard, endoscopic cyclophotocoagulation could achieve a success rate of 76.5% (mean follow-up: 10.8 months) at our center [24], which was significantly better than ultrasonic cyclocoagulation. However, cyclophotocoagulation has serious complications; for example, the incidence of phthisis was about 10% [25]. In contrast, the current HIFU-based ultrasonic cyclocoagulation has its own strengths that it is a relatively noninvasive procedure and is easy to operate, with an acceptable safety profile.

This study had some limitations. First, because of the short duration of follow-up (only 3 months), long-term efficacy need to be further evaluated. Second, the sample size (61 patients) may not be large enough for a convincing analysis of rare complications. As the individuals involved were end-stage glaucoma patients, it was difficult to effectively assess the impact of treatment on visual acuity.

In summary, HIFU-based ultrasonic cyclocoagulation is an effective treatment to reduce IOP for Chinese patients with end-stage glaucoma. It is expected to serve as a promising alternative to cyclodestructive surgery. However, long-term efficacy and safety require further investigation.

## Figures and Tables

**Table 1:** Patient demographics.

Groups	6 sectors	8 sectors	All	*p*
Number of patients	43	18	61	—
Age, mean (range), y	59.2 (27–86)	52.5 (22–77)	57.2 (22–86)	0.130
Sex (male/female)	28/15	6/12	34/27	0.023
Diagnosis (n (%))				
POAG	7 (16.3)	3 (16.7)	10 (16.4)	0.867^*∗*^
PACG	14 (32.6)	4 (22.2)	18 (29.5)	—
NVG	19 (44.2)	10 (55.6)	29 (47.5)	—
Traumatic	3 (7.0)	1 (5.6)	4 (6.6)	—
Glaucoma surgery				0.360^*∗*^
Trabeculectomy	10	2	12	—
AC penetration	1	0	1	—
Vision acuity				
NLP	37	18	55	—
CF-LP	6	0	6	—
Follow-up completed	35/43 (81.4)	17/18 (94.4)	52/61(85.2)	—

POAG: primary open-angle glaucoma; PACG: primary angle closure glaucoma; NVG: neovascular glaucoma; AC: anterior chamber; NLP: no light perception; CF: count finger; LP: light perception. ^*∗*^Fisher's exact test.

**Table 2:** IOP levels at return visits for two groups of patients.

Groups	FU	*N*	IOP (mmHg)	*p* ^&^	IOP range	IOP reduction (%)	Number of medications	*p* ^*∗*^
6 sectors	Baseline	43	40.07 ± 10.18	—	22–59	—	2.6 ± 1.1	—
	D1	41	30.91 ± 10.09	<0.001	11–55	22.20	2.6 ± 1.1	1
	D7	43	25.42 ± 11.14	<0.001	8–48.3	36.60	2.6 ± 1.1	1
	M1	42	28.34 ± 12.92	<0.001	8.3–57.7	29.40	2.6 ± 1.2	0.589
	M3	34	31.65 ± 12.56	<0.001	2–55	22	2.4 ± 1.2	0.34

8 sectors	Baseline	18	43.59 ± 11.62	—	26–64	—	2.7 ± 0.7	—
	D1	18	25.02 ± 9.24	<0.001	5–36	42	2.7 ± 0.7	1
	D7	18	18.46 ± 10.66	<0.001	4.3–36.7	57.70	2.7 ± 0.7	1
	M1	17	22.98 ± 10.15	<0.001	9–40.3	50.20	2.6 ± 0.7	0.317
	M3	17	31.59 ± 18.44	0.019	3–79	28.50	2.3 ± 1.1	0.038

D1: day 1; D7: day 7; M1: month 1; M3: month 3; IOP: intraocular pressure. ^&^Paired *t*-test, compared to baseline IOP; ^*∗*^Wilcoxon's test.

**Table 3:** Subgroup analyses.

Subgroups	*N*	Baseline IOP (mmHg)	IOP reduction at M3 (mmHg)	Percentage of IOP reduction (%)	Success rate		*p*
POAG	9	37.84 ± 9.20	6.69 ± 8.21	17.7	5/9	55.6%	0.013^&^
PACG	15	43.99 ± 11.88	15.90 ± 13.66	36.1	12/15	80.0%	—
NVG	24	41.77 ± 11.11	7.78 ± 17.05	18.6	7/24	29.2%	—
Traumatic	4	39.28 ± 14.21	8.50 ± 15.20	21.6	2/4	50.0%	—

No surgery	40	41.14 ± 11.11	8.98 ± 15.93	21.8	17/40	42.5%	0.100^*∗*^
Surgery	12	42.86 ± 11.66	13.37 ± 10.46	31.2	9/12	75.0%	—

Total	52	41.11 ± 10.65	9.99 ± 14.87	24.3	26/52	50%	—

POAG: primary open-angle glaucoma; PACG: primary angle closure glaucoma; NVG: neovascular glaucoma; IOP: intraocular pressure. ^&^Fisher's exact probability; ^*∗*^chi-squared tests with correction for continuity.

**Table 4:** Complications due to treatment.

Complications	6 sectors (*n*=43)	8 sectors (*n*=18)	Total (*n*=61)
Anterior chamber reaction	13	9	22
Conjunctival hyperemia	10	5	15
Keratic precipitates	9	6	15
Chemosis	8	6	14
Intraoperative pain	10	3	13
Subconjunctival hemorrhage	6	7	13
Scleral thinning	7	5	12
Foreign body sensation	4	1	5
Superficial punctate keratitis	3	1	4
Corneal edema	1	1	2
Hypotony	1	1	2
Astigmatism (>1 diopter)	1	0	1
Retinal detachment^*∗*^	1	0	1

Patients with ocular signs mentioned in the table were included for analyses only if such signs occurred or aggravated after the treatment. ^*∗*^The causality was not confirmed due to severe cataract and no ultrasound scan of the fundus before the treatment.

## Data Availability

The data used to support the findings of this study are available from the corresponding author upon request.

## Supplementary Materials

Supplementary Materials Table S1: IOP change and success rate for different groups. Table S2: visual acuity outcomes for patients with VA better than or equal to LP. Table S3: relevant data for patients suffering from pain. Figure S1: relevant data of the patient who presented retinal detachment at 3 month.
