# Supplementary material for: Short-Term Observation of Ultrasonic Cyclocoagulation in Chinese Patients with End-Stage Refractory Glaucoma: A Retrospective Study
Source: J Ophthalmol. 2018 Sep 6;2018:4950318. doi: 10.1155/2018/4950318 (PMC6148825; doi:10.1155/2018/4950318)
Supplement: Supplementary Materials — Table S1: IOP change and success rate for different groups. Table S2: visual acuity outcomes for patients with VA better than or equal to LP. Table S3: relevant data for patients suffering from pain. Figure S1: relevant data of the patient who presented retinal detachment at 3 month. [file 4950318.f1.zip › 4950318.f1/S.Figure 1.docx]

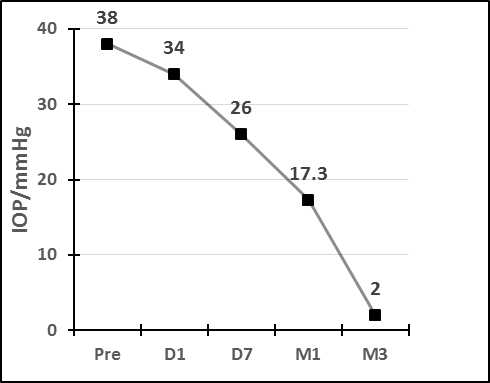

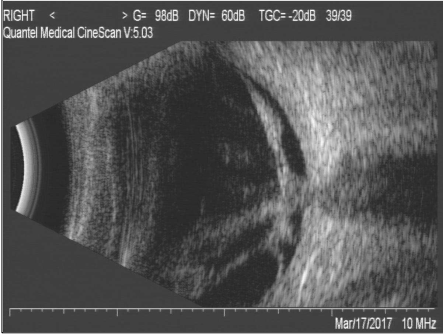


**A B**

**C D**


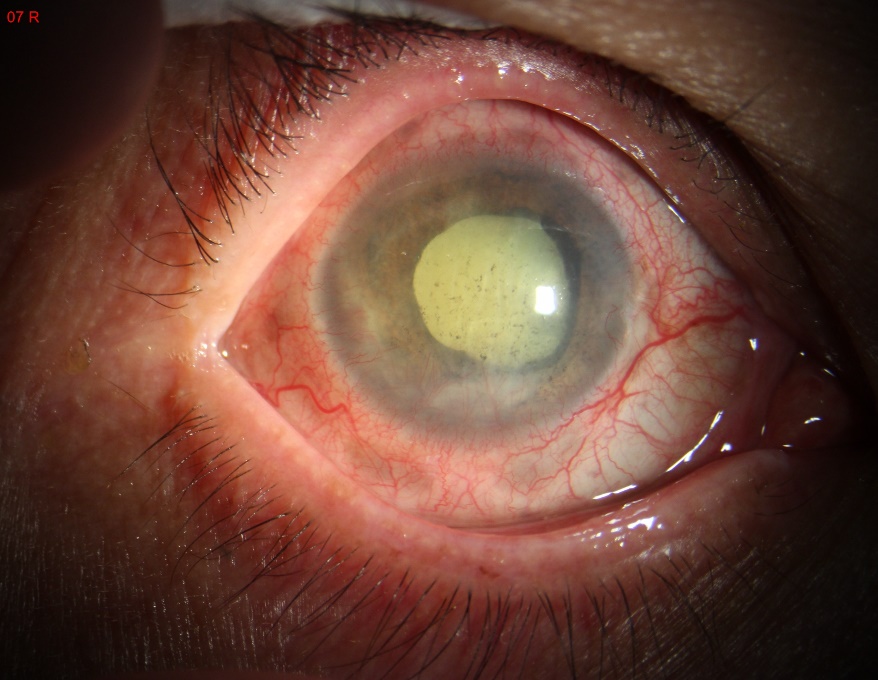

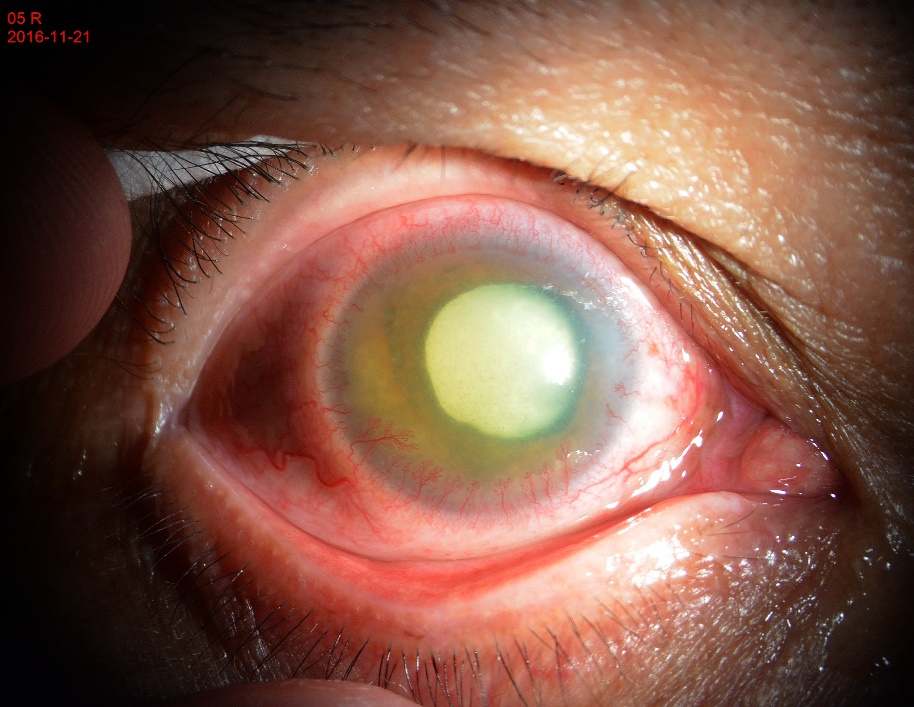


S.Figure 1. Relevant data of the patient who presented retinal detachment at 3 month. A. The baseline picture of ocular surface; B. Anterior segment picture captured at last follow-up; C. IOP trend showed continuing decrease; D. Retinal detachment was detected, accompanied by hypotony (2mmHg) at 3 month.
