# Supplementary material for: Short-Term Observation of Ultrasonic Cyclocoagulation in Chinese Patients with End-Stage Refractory Glaucoma: A Retrospective Study
Source: J Ophthalmol. 2018 Sep 6;2018:4950318. doi: 10.1155/2018/4950318 (PMC6148825; doi:10.1155/2018/4950318)
Supplement: Supplementary Materials — Table S1: IOP change and success rate for different groups. Table S2: visual acuity outcomes for patients with VA better than or equal to LP. Table S3: relevant data for patients suffering from pain. Figure S1: relevant data of the patient who presented retinal detachment at 3 month. [file 4950318.f1.zip › 4950318.f1/Supplementary Materials.docx]

S.Table 1. IOP change and success rate for different groups

| **Sectors** | **Diagnosis** | **IOP** | **N.** | **Mean±SD** | **Range** | **Reduction** | **M3 success proportion** |
| --- | --- | --- | --- | --- | --- | --- | --- |
| 6 sectors | POAG | Baseline | 7 | 36.61±10.65 | 22-52 | - | 4/6, 66.7% |
|  |  | D1 | 6 | 26.72±6.72 | 15-34.3 | 27% |  |
|  |  | D7 | 7 | 22.19±13.04 | 11-46.3 | 39.4% |  |
|  |  | M1 | 7 | 25.81±12.57 | 13.7-49 | 29.5% |  |
|  |  | M3 | 6 | 29.5±9.52 | 16-42 | 19.4% |  |
|  | PACG | Baseline | 14 | 40.89±11.08 | 25-56.7 | - | 8/11, 72.7% |
|  |  | D1 | 14 | 25.9±9.43 | 11-46 | 36.7% |  |
|  |  | D7 | 14 | 23.16±13.17 | 8-48.3 | 43.4% |  |
|  |  | M1 | 13 | 24.78±15.47 | 8.3-57.7 | 39.4% |  |
|  |  | M3 | 11 | 28.82±13.02 | 10-52 | 29.5% |  |
|  | NVG | Baseline | 19 | 40.62±8.82 | 30-59 | - | 3/15, 20% |
|  |  | D1 | 18 | 35.68±8.35 | 24-55 | 12.2% |  |
|  |  | D7 | 19 | 28.86±8.65 | 17.7-45.7 | 29% |  |
|  |  | M1 | 19 | 31.11±11.05 | 12.3-48 | 23.4% |  |
|  |  | M3 | 14 | 35.71±13.73 | 2-55 | 12.1% |  |
|  | Traumatic | Baseline | 3 | 40.8±17 | 23.7-57.7 | - | 2/3, 66.7% |
|  |  | D1 | 3 | 34±17.44 | 14-46 | 16.7% |  |
|  |  | D7 | 3 | 21.7±9.54 | 15.7-32.7 | 46.8% |  |
|  |  | M1 | 3 | 32.1±14.67 | 19.7-48.3 | 21.3% |  |
|  |  | M3 | 3 | 27.33±10.07 | 18-38 | 33% |  |
| 8 sectors | POAG | Baseline | 3 | 38.67±3.21 | 35-41 | - | 1/3, 33.3% |
|  |  | D1 | 3 | 32±3 | 29-35 | 17.2% |  |
|  |  | D7 | 3 | 20.33±4.93 | 17-26 | 47.4% |  |
|  |  | M1 | 3 | 28.77±10.04 | 22-40.3 | 25.6% |  |
|  |  | M3 | 3 | 34.67±8.74 | 25-42 | 10.3% |  |
|  | PACG | Baseline | 4 | 50.93±9.04 | 38.3-59.7 | - | 4/4, 100% |
|  |  | D1 | 4 | 15.5±8.35 | 5-24 | 69.6% |  |
|  |  | D7 | 4 | 9.9±5.54 | 4.3-15 | 80.6% |  |
|  |  | M1 | 4 | 20±13.29 | 9-38 | 60.7% |  |
|  |  | M3 | 4 | 26±11.78 | 14-42 | 48.9% |  |
|  | NVG | Baseline | 10 | 43.03±13.59 | 26-64 | - | 4/9, 44.4% |
|  |  | D1 | 10 | 26.73±8.74 | 9-36 | 37.9% |  |
|  |  | D7 | 10 | 21.17±12.52 | 5.7-36.7 | 50.8% |  |
|  |  | M1 | 9 | 22.67±9.83 | 10-37.7 | 47.3% |  |
|  |  | M3 | 9 | 31.89±24 | 3-79 | 25.9% |  |
|  | Traumatic | Baseline | 1 | - | 34.7 | - | 0/1, 0 |
|  |  | D1 | 1 | - | 25 | 28% |  |
|  |  | D7 | 1 | - | 20 | 42.4% |  |
|  |  | M1 | 1 | - | 20.3 | 41.5% |  |
|  |  | M3 | 1 | - | 42 | -21% |  |

IOP: intraocular pressure, D1: day 1, D7: day 7, M1: month 1, M3: month 3, POAG：primary open angle glaucoma, PACG：primary angle closure glaucoma, NVG：neovascular glaucoma, IOP: intraocular pressure.

S.Table 2. Visual acuity outcomes for patients with VA better than NLP

| N. | Age/ys |  | Pre | D1 | D7 | M1 | M3 | Possible reasons for VA change |
| --- | --- | --- | --- | --- | --- | --- | --- | --- |
| 15 | 27 | VA | CF | - | - | CF | CF | NA |
|  |  | IOP | 29 | 15 | 14 | 13.7 | 30 |  |
| 21 | 68 | VA | CF | - | - | CF | Lost FU | NA |
|  |  | IOP | 44.3 | 35 | 26.3 | 35.3 | Lost FU |  |
| 30 | 63 | VA | HM | - | - | HM | NLP | Uncontrolled IOP |
|  |  | IOP | 44 | 21 | 9 | 29.3 | 34 |  |
| 32 | 58 | VA | HM | - | - | NLP | NLP | Cataract progressed & |
|  |  | IOP | 30 | 36 | 36.3 | 30 | 21 | Uncontrolled IOP |
| 52 | 83 | VA | LP- | - | - | HM | NLP | Uncontrolled IOP |
|  |  | IOP | 41.7 | 40 | 40.3 | 48 | 36 |  |
| 61 | 59 | VA | HM | - | - | HM | HM | NA |
|  |  | IOP | 31.3 | 17 | 14 | 14 | 19 |  |

CF: count finger, HM: hand movement, LP: light perception, NLP: no light perception

S.Table 3. Relevant data for patients suffering from pain

| FU | Pre | D1 | D7 | M1 | M3 |
| --- | --- | --- | --- | --- | --- |
| Num. | 14 | 21 | 12 | 4 | 1 |
| Mean PS | 4.5 | 3.8 | 3.1 | 2.75 | 6 |
| Mean IOP/mmHg | 40.2 | 31.0 | 25.0 | 27.8 | 18 |
| Cornea edema/neural pain | 8/6 | 11/10 | 4/8 | 1/3 | 0/1 |

FU: follow-up, PS: pain score, IOP: intraocular pressure

.
